# Beneath the cuff: Often overlooked and under-reported blood flow restriction device features and their potential impact on practice—A review of the current state of the research

**DOI:** 10.3389/fphys.2023.1089065

**Published:** 2023-03-30

**Authors:** Nicholas Rolnick, Kyle Kimbrell, Victor de Queiros

**Affiliations:** ^1^ The Human Performance Mechanic, CUNY Lehman College, NY, United States; ^2^ Owens Recovery Science, San Antonio, TX, United States; ^3^ Graduate Program in Health Sciences, Federal University of Rio Grande do Norte (UFRN), NatalRN, Brazil

**Keywords:** safety, autoregulation, bladder, kaatsu, occlusion training, BFR training

## Abstract

Training with blood flow restriction (BFR) has been shown to be a useful technique to improve muscle hypertrophy, muscle strength and a host of other physiological benefits in both healthy and clinical populations using low intensities [20%–30% 1-repetition maximum (1RM) or <50% maximum oxygen uptake (VO_2max_)]. However, as BFR training is gaining popularity in both practice and research, there is a lack of awareness for potentially important design characteristics and features associated with BFR cuff application that may impact the acute and longitudinal responses to training as well as the safety profile of BFR exercise. While cuff width and cuff material have been somewhat addressed in the literature, other cuff design and features have received less attention. This manuscript highlights additional cuff design and features and hypothesizes on their potential to impact the response and safety profile of BFR. Features including the presence of autoregulation during exercise, the type of bladder system used, the shape of the cuff, the set pressure *versus* the interface pressure, and the bladder length will be addressed as these variables have the potential to alter the responses to BFR training. As more devices enter the marketplace for consumer purchase, investigations specifically looking at their impact is warranted. We propose numerous avenues for future research to help shape the practice of BFR that may ultimately enhance efficacy and safety using a variety of BFR technologies.

## 1 Introduction

The interest and adoption of blood flow restriction (BFR) training in the rehabilitation and fitness settings has increased substantially in recent years (de Queiros et al., 2021; Mills et al., 2021; Cuffe et al., 2022). Fueled at least in part by its ability to generate musculoskeletal and cardiovascular performance benefits with reduced mechanical loads (Lixandrão et al., 2018; Formiga et al., 2020), this interest has also fueled an in-kind response from the device manufacturing market. As is true of all product markets, manufacturers have included a variety of different features to their respective BFR cuffs to appeal to the consumer. Apart from cuff width, little is known regarding most features that make one cuff different from the next. Device features that impact the delivery of pressure to the limb are of particular importance given pressure’s impact on acute responses to the exercise technique (Hughes et al., 2018; Jacobs et al., 2023). These acute responses will have safety implications due to their impact on hemodynamics but may also have longitudinal influence given the associated perceptual responses (Rossow et al., 2012).

To the authors’ knowledge, Loenneke et al. (Loenneke et al., 2012) was the first to suggest that applied BFR pressures be standardized relative to the cuff and the individual using arterial occlusion pressure (AOP). AOP is the minimum pressure needed to completely occlude arterial inflow and venous return to the limb (Patterson et al., 2019). The use of AOP has its roots in the surgical world where it has been suggested both safety and post-operative pain are affected; increased use in the BFR literature has been both encouraged by a group of experienced researchers and reported in recent reviews and trials (AORN Recommended Practices Committee, 2007; Morehouse et al., 2021; Murray et al., 2021). This approach inherently controls for variances in cuff widths (Mouser et al., 2018; Evin et al., 2021), limb circumferences and participant blood pressures (Loenneke et al., 2015). Left uncontrolled, other applied pressure schemes common in the BFR literature (e.g., 200 mmHg, 1.3x systolic blood pressure, etc.) may unfavorably impact acute responses to BFR exercise, reducing adherence while increasing the potential for exercise to be carried out under full occlusion (Chulvi-Medrano et al., 2023). Moreover, the use of personalized pressures prescribed as a %AOP may reduce the heterogeneity observed in the systematic reviews and meta-analyses (Lixandrão et al., 2018; Clarkson et al., 2019; Formiga et al., 2020; Grønfeldt et al., 2020), leading to more precise estimates on the magnitude of the effects of BFR exercise and a greater ability to generalize research findings to practice.

For example, one study compared the acute muscular and perceptual responses to a bout of four sets of biceps curl performed with either a 3 cm wide Kaatsu^®^ (Kaatsu Master, Sato Sports Plaza, Tokyo Japan) elastic cuff inflated to an arbitrary 160 mmHg applied pressure or a 5 cm wide Hokanson (Hokanson, Bellevue, WA, United States) nylon cuff inflated to 40% AOP (Dankel et al., 2017). Despite similar cellular swelling, electromyographic amplitudes and post-exercise torque production, the nylon cuff condition reported greater number of repetitions performed during sets 2 and 3, lower rate of perceived exertion during set one and lower rate of perceived discomfort during all sets compared to the elastic cuff condition. The discrepancy between conditions in perceptual responses and repetitions to failure may be explained by the higher relative applied pressure of the elastic cuff (∼65 ± 19% AOP) compared to the nylon cuff (40% AOP). Giving further support, when cuffs of different widths and materials are standardized to a %AOP, the physiologic and perceptual responses are largely equivocal (Loenneke et al., 2012; Loenneke et al., 2013; Buckner et al., 2017) indicating that much of the differences observed following arbitrary pressure application protocols are likely due to varied degrees of relative personalized pressures.

BFR cuff systems marketed to consumers may possess modifications in shape, bladder construction, pressure control, material qualities or the ability to adjust pressure in response to contracting muscle that likely affect the delivery of pressure to the limb and/or ability to determine a personalized pressure. However, many of these device features have little to no evidence the end user can reference to support prioritizing certain features over others. The purpose of this manuscript is to expound upon these cuff features, reviewing what evidence we possess and illustrating the importance of continued empirical investigations into these features so that practitioners can make informed decisions and device manufacturers can continue to innovate.

## 2 The impact of lesser-known cuff features on personalized pressure application and responses to BFR exercise

### 2.1 Multi-vs. Single-chambered bladder system

A tourniquet—by definition—is designed to occlude arterial flow (Noordin et al., 2009). This function forms the basis for the majority of BFR cuffs on the marketplace and in research because it allows a personalization of applied pressure with additional technology (e.g., doppler ultrasound, pulse pressor sensors, etc.) (Patterson et al., 2019). The bladder in a single-chambered system completely encircles the limb and inflates with air to apply pressure to the limb. Studies indicate that these types of bladder systems interact with cardiovascular and perceptual responses which may impact tolerability or the potential safety of the approach (Rossow et al., 2012), while a few studies note attenuated hypertrophy of the muscles underneath the cuff (Kacin and Strazar, 2011; Ellefsen et al., 2015). However, the attenuated hypertrophy underneath the cuff observed in some studies appears to not occur when pressure is personalized using %AOP (Laurentino et al., 2016).

**FIGURE 1:**
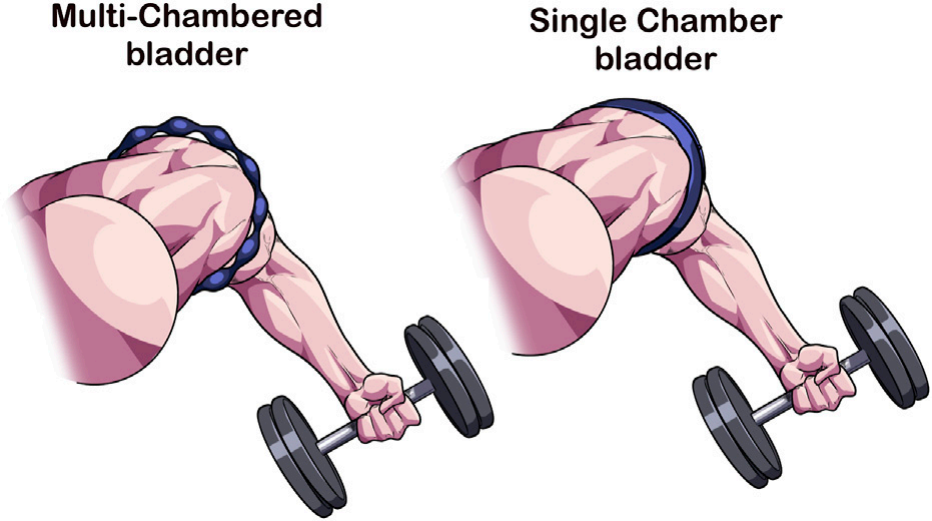
Multi-chambered versus single-chambered bladder cuff design. As opposed to traditional tourniquets whose function is to occlude arterial flow, multi-chambered bladders are composed of sequential bladders that when inflated, leave regions where minimal compression occurs. This cuff feature reduces the ability for the device to occlude arterial flow making it difficult to obtain a personalized pressure. The inability to occlude has been hypothesized to enhance safety during BFR exercise.

Recently, commercially available devices have entered the marketplace that consist of numerous sequential bladders that according to the manufacturer (B-Strong™ Bands (B-Strong Training Systems™, Park City, UT, United States); B3 BFR Bands (B3 Sciences, Frisco, TX, United States) are designed to reduce the potential for arterial occlusion and may result in a non-uniform circumferential pressure during exercise (Figure 1) (Early et al., 2020). As the multi-chambered bladder system is not designed to occlude, measurement of AOP is largely unfeasible in most individuals (Citherlet et al., 2022), so the manufacturer recommends pressures of 250 mmHg for the upper body and 350 mmHg for the lower body (Early et al., 2020) or a pressure based on individual factors using an app (Bordessa et al., 2021; Callanan et al., 2022). These cuff systems do appear to induce additional acute physiological stress over work-matched free flow exercise (Stray-Gundersen et al., 2020; Wooten et al., 2020), indicating that they have the potential to produce beneficial adaptations in a longitudinal training approach. However, as noted earlier, it is difficult to personalize pressure relative to the individual with this cuff design. Not personalizing the pressure may alter magnitude of the BFR stimulus and subsequent adaptation profile.

To the authors’ knowledge, only three training studies utilizing a multi-chambered bladder system have been published and none of those studies were constructed in a manner that the results might elucidate the potential efficacy of the bladder type with respect to blood flow restricted exercise (Table 1). We will discuss each of these studies briefly to inform the reader of the state of the body of research and comment on their potential implications to our understanding of BFR exercise with multi-chambered bladder cuff systems.

**TABLE 1:** Summary of available acute and chronic blood flow restriction training studies using a multi-chambered bladder system.

**Reference**	**Sample (M/F)**	**Study design**	**Duration (Weekly frequency)**	**Intensity**	**Volume**	**Device (cuff width)**	**Pressure**	**Outcomes**	**Conclusions**
**Chronic studies**									
Early et al. (2020)	31 healthy adults (11/20)	Randomized controlled trial (Between-subjects)	8 weeks (2-3)	LL-BFR: 30-50% 1-RM	LL-BFR: 3x10 reps	B-Strong™ Bands (Arms: 5.5 cm/Legs: 7.0 cm)	Arms: 250 mmHg	Artery diameter (FMD)	There was no significant difference between interventions for strength, endurance, or cardiovascular outcomes. Pain was lower in B-Strong™ bands after the last session.
				HL-RT: 60-100% 1-RM	HL-RT: 3x30 reps		Legs: 350 mmHg	Blood pressure	
								Pain	
								Muscle strength	
								Muscle endurance	
Wang et al. (2022)	18 collegiate volleyball players (18/0)	Randomized controlled trial (Between-subjects)	8 weeks (3)	LL-BFR: 30% 1-RM	LL-BFR: 4x10 reps	B-Strong™ Bands (7.0 cm)	50% estimated AOP based on thigh circumference	Muscle strength	For muscle strength, there were significant differences between groups, favoring high-load interventions. Only HL-BFR promoted improved jumping performance.
				HL-BFR: 70% 1-RM	HL-RT: 4x8 reps			Vertical jump	
				HL-RT: 70% 1-RM					
Wooten et al. (2022)	21 older patients with abdominal cancer	Cohort	4 weeks (5-6)	NR	NR	B-Strong™ Bands (NR)	NR	Length of hospital stay	BFR training plus sports nutrition supplementation was effective in reducing postoperative complications and length of hospital stay.
								Postoperative complications	
								Readmission rate	
								Mortality at 90 days post-surgery	
**Acute studies**									
Bordessa et al. (2021)	34 healthy adults (18/16)	Crossover randomized trial	—	LL-BFR: 30% 1-RM	LL-BFR: 1x30 + 3x15	B-StrongTM Bands (5.0 cm)	250-310 mm Hg 80% AOP	RPE	Resistance exercise with B-Strong™ bands induced less pronounced perceptual responses.
				HL-RT: 80% 1-RM	HL-RT: 2x8 + 2x6	Delfi Personalized Tourniquet System (11.5 cm)		Pain	
								EMG	
Callanan et al. (2022)	15 healthy adults (8/7)	Crossover randomized trial	—	NR for both free-flow and BFR conditions	3x3 minutes of VersaClimber	BStrong™ Bands (Arms: 5.0 cm/Legs: 7.5 cm)	NR	WBC	RPE significantly greater in B-Strong™ bands above free-flow and both groups improved in WBC, platelets, lymphocytes, CD34+ and blood glucose and decreased peripheral neutrophils post-exercise. No significant differences between groups.
								Platelets	
								Neutrophils	
								Lymphocytes	
								CD34+	
								Lactate	
								Blood glucose	
								RPE	
Citherlet et al. (2022)	11 healthy adults (4/7)	Crossover randomized trial	—	—	—	B-Strong™ Bands (Arms: 5.0 cm/Legs: 7.5 cm)	200, 250, 300, 350, and 400 mmHg 0, 40 and 60% AOP	Blood flow	Both devices were able to reduce blood flow. For B-Strong™ bands, this response was achieved only with 350 and 400 mmHg. With Hokanson, the two pressures tested were able to reduce blood flow.
						E20 rapid cuff inflator, Hokanson (Arms: 5 cm; Legs: 10 cm)			
Machek et al. (2022)	18 recreationally active adults (18/0)	Crossover randomized trial	—	LL-BFR: 20% 1-RM	LL-BFR: 1x30 + 3x15 + 2 sets to failure	B3 Bands (9.525 cm)	80% AOP	RPE	Betaine supplementation 14 days prior did not provide additive benefit to outcomes in any loading condition. However, HL-RT increased lactate concentrations post-exercise over B3 Bands condition and B3 Bands condition had higher RPE and discomfort than HL-RT.
				HL-RT: 70% 1-RM	HL-RT: 4x10 + 2 sets to failure			Discomfort	
								Lactate	
								Serum GH, IGF-1, and HCY	
Stray-Gundersen et al. (2020)	15 healthy adults (9/6)	Crossover randomized trial	—	0.9 m/s (Walking)	5x2 minutes	B-Strong™ Bands (5 cm)	160 mmHg	Blood lactate	Walking with B-Strong™ bands induced less pronounced responses than Hokanson cuff.
						E20 rapid cuff inflator, Hokanson (18 cm)	300 mmHg	RPE	
								Blood pressure	
								Heart rate	
								Arterial stiffness	
Wilburn et al. (2021)	1 healthy adult (1/0)	Case report	—	LL-BFR: 30% 1-RM	LL-BFR: 1x30 + 3x15 + 2 sets to failure	B3 Bands (9.525 cm)	80% AOP	Muscle damage	Muscle damage appeared to be elevated in the HL-RT condition compared to B3 Bands condition. Sarcomere orientation and sarcomere length differed post-exercise, with wave-like orientation and intracellular abnormalities observed in B3 Bands condition.
				HL-RT: 70% 1-RM	HL-RT: 4x10 + 2 sets to failure			Sarcomere orientation	
								Sarcomere area	
								Sarcomere lengh	
								M-band width (all via electron micrograph)	
Wooten et al. (2020)	20 healthy adults (10/10	Crossover randomized trial	—	20 Yoga poses	LL-BFR: N/R	B-Strong™ Bands (6 cm arms; 6 cm legs)	300 mm Hg	RPE	No significant differences between conditions were observed except for a greater increase in lactate levels in the B-Strong™ cuff condition.
					LL: N/R			CAVI	
								Blood pressure	
								HR	
								Double product	
								FMD	
								Lactate	

1-RM, 1 repetition maximum; AOP, arterial occlusion pressure; BFR, blood flow restriction; EMG, electromyography; F, female; FMD, flow-mediated dilation, HL, high-load; LL, low-load; RPE, rate of perceived exertion; NR, not reported; WBC, white blood cell; GH, Growth hormone; IGF-1, Insulin-like growth factor 1; HCY, Homocysteine; CAVI, cardio-ankle vascular index; HR, heart rate

Early et al. (Early et al., 2020) compared the muscular performance, pain and vascular function following 8 weeks of BFR exercise performed with the B-Strong™ cuff in 31 healthy participants (n = 20 females). They randomized participants to either a traditional resistance exercise (3 × 10 repetitions at 60% 1RM), BFR exercise (3 × 30 repetitions or to failure at 30% 1RM), or a non-exercise control group. Participants in the exercise groups performed 20 exercise sessions (2–3x/week) over the training period with seven upper and lower body exercises performed in each session. Load was progressed 10% every second week in both groups, but the BFR group was capped at 50% 1RM. Applied pressure in the BFR condition ranged between 250 mmHg in the upper body to 350 mmHg in the lower body and was kept on continuously and deflated only when changing from upper body to lower body exercise. Results showed that after 8 weeks, BFR was able to elicit similar vascular adaptations (evidenced by small [∼0.5–1%] improvements in flow mediated dilatation; *p* = 0.006) and strength gains as traditional resistance exercise in all 1RM tests (*p* > 0.05) with less perceived muscle pain (evidenced by the visual analog scale) during the last session (*p* < 0.05). The conclusions support the use of a multi-chambered bladder system to induce comparable physiological changes as traditional resistance exercise. However, as the study design had some participants exercise to failure in the BFR group and did not include a low load control group or monitor volume load (reps x sets x load) between groups, it is difficult to surmise any potential impact of the bladder system on the outcomes of the study. This is relevant to the discussion of bladder type because low-load exercise with- and without BFR to muscular fatigue has been shown to improve muscle mass and strength to a similar degree (Fahs et al., 2015; Pignanelli et al., 2020). To date, no study has investigated longitudinal musculoskeletal outcomes and tracked volume load when exercise is performed to failure between low-loads with and without different BFR bladder designs and high loads (>70% 1-repetition maximum).

The second study analyzed the benefits of a 4-week multi-modal prehabilitation program combining exercise with B-Strong™ bands and a sports nutrition supplement in 21 patients with abdominal cancer (Wooten et al., 2022). While the results of the study indicated a beneficial effect on reducing complications (*p* = 0.03) and length of hospital stay (∼5.5 fewer days, *p* = 0.02) as well as a 58% increase in step count 5-day post-op (*p* = −.043), the comparison group was retrospectively analyzed (n = 71) and underwent usual standard of care without BFR or sports supplementation. Therefore, the study design was unable to determine whether the positive impact of the trial was due to the inclusion of the B-Strong™ cuffs, the sports nutrition supplement, or a combination of both.

The third and most recently published study (Wang et al., 2022) investigated the impact of backsquat exercise performed with BFR on performance and muscular strength following 8 weeks of 3x/week training in male resistance-trained volleyball players (n = 18; ∼20 years old). Three experimental groups (n = 6 per group) were randomly formed: low-load BFR performed with 30% 1RM, high load strength training with 70% 1RM, and high load strength training with BFR using 70% 1RM. BFR was applied to the bilateral thighs using B-Strong™ cuffs at 50% estimated arterial occlusion pressure and was on continuously in all BFR conditions (e.g., was inflated before the exercise and released after the exercise only). The low-load BFR group exercised with the commonly recommended BFR fixed repetition scheme of 30–15–15–15 with 60 s of interset rest whereas the high load strength training with- and without BFR was done for four sets of eight repetitions with 60 s of interset rest. After 8 weeks (24 sessions), max backsquat strength improved for all groups compared to baseline, but the high load strength training group with- (28.6%; *p* = 0.00) and without BFR (17.3%; *p* = 0.003) improved more (*p* = 0.19) than the low-load BFR group (9.9%; *p* = 0.001). Additional muscle strength results measuring peak isokinetic knee flexion and extension torques (at 60°/s) exhibited a similar trend. The high load strength training groups with- and without BFR improved peak knee extension (between 11.7%–17.7%, *p* < 0.01–*p* < 0.05) and flexion (10.9%–16.5%, *p* < 0.01–*p* < 0.05) torques to a greater degree (*p* = 0.005–0.048) over low-load BFR (between 5.1%–8.7% in both muscle groups) with no between-group differences (*p* > 0.05). Last, jump performance as assessed by a squat jump and three-footed takeoff test improved only in the high load strength training group with BFR (*p* = 0.015–*p* = 0.02) with significantly larger improvements than the low load BFR group (*p* = 0.002–0.039). The results of this study support that BFR using the B-Strong™ cuffs with high load strength training to maximize muscle strength and jump performance in trained athletes. Of note, BFR exercise has been recommended to be performed with low-intensity exercise (e.g., 20%–40% 1RM or <50% VO_2_max) (Patterson et al., 2019), so the results of this study challenge current recommendations for practice. Further, as hemodynamics were not assessed in the study design, it is not known the impact of exercising with BFR using heavier loads and possible safety risk. This is particularly relevant given the current body of evidence showing that hemodynamic responses are predominantly driven by load lifted (MacDougall et al., 1985; Sale et al., 1994) and also by the application of BFR (Domingos and Polito, 2018).

This study highlights an important reason why this manuscript is being written. The authors attempted to apply BFR at 50% estimated AOP using an algorithm based on thigh circumference, but did not consider that the algorithm was created in reference to single-chambered bladder nylon and elastic BFR systems (Loenneke et al., 2012). Thus, it is likely not valid for use in a multi-chambered bladder system such as the B-Strong™ cuffs. Prior research has shown that the addition of blood flow restriction to high load strength exercise does not augment muscular activation (Dankel et al., 2018; Teixeira et al., 2018) or produce superior muscular hypertrophy or strength compared to the same exercise performed without BFR in single-chambered bladder systems (Laurentino et al., 2008). Of note, the longitudinal study (Laurentino et al., 2008) performed exercise at 100% AOP, had BFR applied intermittently (e.g., released during the rest period), and used loads between six- and 12-RM; so differences do exist between studies that limit strong comparisons. Nonetheless, the misapplication of the limb circumference algorithm in the current study could lead to misinterpretations regarding the effectiveness of BFR using heavier loading schemes with single-chamber bladder BFR cuff systems. Future studies should take care to apply algorithms designed for single-chambered bladder systems in investigations where single-chambered bladder systems are used to avoid potentially compromised study designs and conclusions.

Within the current BFR body of literature, there are three published studies that compared the acute responses of a multi-chambered bladder system to a single-bladder system (Stray-Gundersen et al., 2020; Bordessa et al., 2021; Citherlet et al., 2022) and four studies on multi-chambered bladder systems compared to a free-flow control (Wooten et al., 2020; Wilburn et al., 2021; Callanan et al., 2022; Machek et al., 2022) (Table 1). For completion’s sake, we have displayed the four additional multi-chambered bladder investigations that compared responses to free-flow exercise to highlight the limited overall body of research in this area (Table 1).

All studies save two (Wilburn et al., 2021; Machek et al., 2022) have similar methodological issues due to the multi-chambered cuff construction preventing researchers from making pressures relative to that induced by the single chamber systems. Presumably, this results in a greater magnitude of AOP achieved by the single chambered systems in comparison studies, affecting acute cardiovascular, neuromuscular, and perceptual measures, leading to potentially faulty conclusions on safety risk and/or longitudinal outcomes.

Only two (Bordessa et al., 2021; Citherlet et al., 2022) of the three comparison studies set pressures in the single-chambered system relative to %AOP in the comparison condition, whereas the other study (Stray-Gundersen et al., 2020) assigned an arbitrary pressure. Despite the limitation mentioned, all three studies provide important context to the discussion of the potential impact of bladder design on the BFR stimulus.

The first published comparison study between different BFR cuff bladder designs compared the acute perceptual and hemodynamic responses between the B-Strong™ cuff (5-cm cuff width) and Hokanson rapid-inflator research device (Hokanson, Bellevue, WA, United States) (18-cm cuff width) inflated to 300 mmHg and 160 mmHg, respectively (Stray-Gundersen et al., 2020). The results support the use of the B-Strong™ cuff for BFR walking aerobic exercise as the Hokanson device promoted greater increases in heart rate, blood pressure, and double product during exercise with elevated perceptual demands (all measures *p* < 0.05). Lactate levels were observed to be significantly greater in the Hokanson condition as well, indicating that metabolic stress was likely greater than in the B-Strong™ condition, given that exercise-induced increases in lactate can be an indirect marker for signaling cell metabolic conditions that may induce metabolic acidosis (Robergs et al., 2004). This possibly resulted in a larger stimulation of the afferents governing the muscle metaboreflex response, increasing cardiovascular and perceptual responses (Boushel, 2010). Considering the width of the Hokanson cuff (18 cm), the magnitude of pressure used (160 mmHg), and the demographics of the participants, the authors of this manuscript conjecture that most were exercising very near 100% AOP. For comparison, Hughes et al. (Hughes et al., 2018) used a narrower Hokanson cuff (13 cm v 18 cm width) and reported full arterial occlusion in 18 subjects at 163.33 ± 17.06 mmHg (Hughes et al., 2018). The Hughes et al. cohort likely had higher AOP values than the Stray-Gunderson et al. cohort given the subject pool was entirely male, had higher BMI values (23 ± 3 vs. 28.94 ± 3.28), and higher resting systolic blood pressure (116 ± 11 mmHg vs. 129 ± 9 mmHg), all factors that have been shown *via* direct or indirect evidence to influence AOP.

The second publication compared two commercially available BFR devices [B-Strong™ and Delfi Personalized Tourniquet device (Delfi Medical Innovations^®^, Vancouver, BC, Canada)] at 30% 1RM against a high load strength training control group performed at 80% 1RM. Using a within-subjects design (n = 34; 18 males), muscle excitation and training-related rate of perceived exertion and muscle pain in a fixed repetition (e.g., 30–15–15–15) design during a leg press exercise was assessed (Bordessa et al., 2021). The B-Strong™ cuff was inflated to between 250–310 mmHg based upon participant characteristics while the Delfi Personalized Tourniquet device was inflated to 80% AOP (between 104–208 mmHg), the maximum recommended pressure for practical use (Patterson et al., 2019). Results show similar muscle activation (as evidenced by electromyography) between cuffs conditions (*p* > 0.05), but both were less than the high load exercise condition (*p* < 0.01). In addition, the B-Strong™ cuff elicited significantly less discomfort (*p* < 0.001) and perceptual exertion (*p* < 0.001) than the Delfi Personalized Tourniquet device condition and were greater than the high load strength condition (*p* < 0.001). As the Delfi Personalized Tourniquet device is a single-chambered bladder tourniquet (Weatherholt et al., 2019), the exercisers in this trial were likely experiencing a greater magnitude of muscle fatigue and were probably significantly closer to failure than those exercising in the B-Strong™ cuff trial given the B-Strong™ cuffs are not designed to occlude blood flow (Early et al., 2020). Research has shown that proximity to failure augments the perceptual responses experienced (Santos et al., 2021), so it is likely that the higher applied pressures in the Delfi Personalized Tourniquet device trial augmented muscle pain and perceived exertion during exercise. As such, the study’s conclusions stated that B-Strong™ was more tolerable than the Delfi Personalized Tourniquet device while providing similar electromyographic activation of the quadriceps. Practitioners may assume from the study that the B-Strong™ cuff is just as effective as the Delfi Personalized Tourniquet device in a longitudinal program with better participant tolerability and similar muscle activation given the acute responses observed. However, without considering the impact of each cuff on occlusive capabilities and subsequent fatigue accumulation during exercise, extrapolating effectiveness should be done with caution. As accelerated muscle fatigue is likely the primary way BFR induces its beneficial effect on muscle (Jessee et al., 2018), the design of Bordessa et al. (Bordessa et al., 2021) gives limited guidance to the potential efficacy of the B-Strong™ cuff bladder system compared to the Delfi Personalized Tourniquet device as both exercised in a work-matched fashion, limiting our understanding of the proximity to failure between conditions and related perceptual factors. Future research comparing the two bladder types during exercise could include repetitions to momentary muscular failure anchored with a low-load free-flow group. This design could help practitioners understand the magnitude of muscle fatigue induced by the different bladders as evidenced by repetitions to momentary muscular failure in each condition. Similarly, longitudinal work-matched, non-failure training studies can help shed light on the adaptation profiles (e.g., muscle mass and strength) that can help form practical recommendations, particularly if adaptations are similar with lower exercise-induced discomfort in multi-chambered systems.

The most recent study published in late 2022 compared the B-Strong™ cuff to the Hokanson research tourniquet on capacity to modulate resting limb blood flow in the upper and lower limbs (Citherlet et al., 2022). Eleven healthy participants (n = 7 females) had all their extremities assessed with both cuffs and their resting blood flow monitored following application of different pressures (e.g., 40%–60% AOP with Hokanson and 200–400 mmHg pressures with B-Strong™). The authors noted that AOP was unable to be determined in any individual with the B-Strong™ cuff and that resting blood flow was only slightly altered from resting conditions at 350 mmHg (*p* = 0.016, d = 0.688) and 400 mmHg (*p* = 0.002, d = 0.805). Conversely, the Hokanson cuff was able to modulate blood flow from rest in both the 40% AOP (*p* = 0.009, d = 0.715) and 60% AOP (*p* < 0.001, d = 0.948) conditions using pressures between 83–125 mmHg. However, both cuffs displayed an inability to regulate blood flow according to the pressure applied (e.g., exhibiting a direct negative linear relationship with increasing pressure), although this observation was more evident with the B-Strong™ cuff (*p* > 0.05). The results of this study indicate that even at the highest pressures, the multi-chambered bladder system cannot effectively modulate limb blood flow whereas a single-chambered bladder system applied at the lowest minimal recommended pressure can modulate blood flow.

Lastly, although not a direct comparison to other BFR cuff types, Callanan et al. (Callanan et al., 2022) sought to examine the systemic hematopoetic stem cell response to an acute bout of lower extremity exercise using B-Strong™ cuffs. This lab has published previous work demonstrating exercise with Delfi Personalized Tourniquet device as well as Vasper system (Vasper Systems, Mountain View, CA) elicits significant increases in platelets, lactate and hematopoetic stem cells (Callanan et al., 2021a; Callanan et al., 2021b). The authors hypothesize this increase in hematopoetic stem cell response may have clinical utility to ensure a more uniform quality of orthobiologic injections. Thus, determining whether a device like B-Strong™ can achieve a similar result is important. To test this, subjects exercised intensely on a VersaClimber for 9 min while wearing the B-Strong™ cuffs on all four limbs at the manufacturer’s recommended pressures based on anthropometric data and sex of the participant (pressures not described in text). Interestingly, while significant increases in platelets, lymphocytes, CD34^+^ cells, and white blood cells were observed, the B-Strong™ condition did not elicit a response that was different than the free flow condition. This is a departure from the results of the other studies performed by the same author group (Callanan et al., 2021a; Callanan et al., 2021b). However, some of the discrepancy in outcomes between studies could be explained by the differences in the amount of volume performed in each of the exercise protocols.

While it does not appear the occlusion of arterial flow is a mandatory aspect of BFR application, reduction of arterial inflow is believed to be important for reducing oxygen delivery, promoting earlier type 2 muscle fiber recruitment, and accelerating muscular fatigue and metabolic stress (Jessee et al., 2018). Currently, no research exists on comparative effectiveness between bladder types in a longitudinal program. The current body of BFR research indicates that higher applied pressures—at least 50% AOP in the lower extremities–are needed to accelerate the fatigue response beyond that of low-load training (Cerqueira et al., 2021). Devices unable to determine a personalized pressure above 40%–50% AOP (Counts et al., 2016; Cerqueira et al., 2021) risk not applying enough pressure to the limb to elicit a fatiguing stimulus beyond that provided with low-load training alone, potentially leading to conclusions that may contradict the existing body of research using single-chambered bladder systems. Particularly susceptible to this issue are studies that exercise participants in a non-failure, work-matched fashion where proximity to failure is not known. Therefore, cuffs that are unable to occlude arterial inflow to determine a personalized pressure may present difficulties in studies when compared to a personalized pressure application.

To reduce flaws in comparisons between devices with different bladders, future studies should investigate the magnitude of post-exercise muscle fatigue (e.g., isometric/dynamic torque loss) following various application parameters. Of most value to practice are acute studies that compare repetitions to failure between different bladder types applied at recommended application settings (e.g., 250/350 mmHg in multi-chambered bladder systems and 40%–80% AOP in single-bladder systems) and longitudinal studies that track volume load, relevant outcomes, and occurrence of adverse events in non-failure and failure repetition schemes. These experimental designs will greatly increase practical relevancy, thus helping practitioners make informed decisions regarding the device they choose to use with their clients and patients.

### 2.2 Autoregulation of applied pressure

**FIGURE 2:**
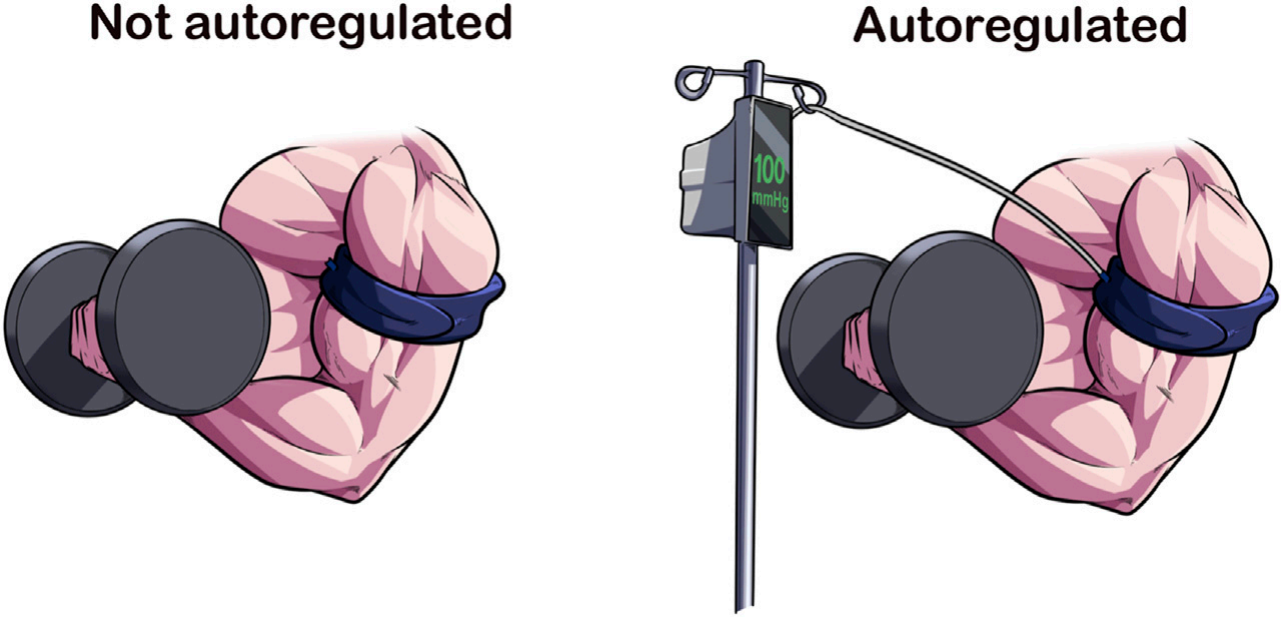
Autoregulation of Applied Pressures. Autoregulation is a design feature that accommodates for the changes in limb circumference because of muscular contraction. In current available devices, the BFR cuff is attached to a pneumatic air compressor *via* an air tubing that adjusts according to the pressure sensed at the cuff-limb interface. The speed at which this adjustment occurs varies across devices, making it a cuff-specific feature. Autoregulation may enhance the acute safety of BFR exercise.

Autoregulation refers to the capability of a device to adjust pressure within the cuff during an inflation cycle. In theory, the result is a more consistent application of pressure to an exercising limb as muscular contractions against the cuff will create spikes in pressure, potentially affecting comfort, hemodynamics, and causing air to escape, possibly reducing the pressure in the cuff for the rest of the session (Kacin et al., 2015; Hughes et al., 2018) (Figure 2). Manual pneumatic cuffs (e.g., non-autoregulated) do not automatically adjust pressure during the inflation cycle; although the user may add pressure lost back into the cuff *via* sphygmomanometer. Until recently, all but one BFR device on the market would be classified as non-autoregulated. Now with multiple devices in the space possessing autoregulation technology, the responsiveness, or speed with which the device can sense and adjust pressure, becomes an important variable in assessing the impact of the feature. Therefore, whether a BFR device has an autoregulation feature, and how that feature performs may be an important variable to report and examine in future investigations.

Currently, Jacobs et al. (Jacobs et al., 2023) is the only study that has directly investigated autoregulation as a primary variable in a within-subjects research design. Using a cohort of 56 healthy, physically active men and women they compared the acute cardiovascular, perceptual, and performance outcomes during a 20% 1-RM leg extension exercise performed at fixed and failure repetition schemes with- and without autoregulation of applied pressures using an identical width (10.16 cm) Smartcuffs™ BFR cuff (Smart Tools Plus LLC, Strongsville, OH, United States). Exercise was performed at 60% AOP (determined in sitting) with a 4 s cadence (2 s concentric/2 s eccentric) per repetition. They also monitored for the occurrence of adverse responses to BFR exercise. Their results showed a 3x risk reduction in the odds of experiencing a minor adverse event (e.g., lightheadedness) compared to the non-autoregulated condition. In addition, during four sets of exercise to failure, the autoregulated condition performed significantly more volume than the non-autoregulated condition (∼199 reps vs. ∼161 reps, *p* < 0.001) with less delayed onset muscle soreness [3 ± 2.2 vs. 4 ± 2.6, *p* < 0.001; 95% confidence interval (CI): 0.544–1.022] and similar blood pressure responses. Though small, they also noted that autoregulation appeared to reduce the perceptual demands during both repetition schemes (*p* < 0.028–<0.001). Thus, results indicate a beneficial impact of the autoregulation feature of the Smartcuffs™ BFR cuff system.

A recent preprint provided additional context to the discussion of autoregulation of applied pressures during BFR exercise. In another within-subject design, Rolnick et al. (Rolnick et al., 2023) investigated the acute central stiffness and muscle morphological responses to four sets of exhaustive wall squat exercise. Squats were performed at 20% 1-RM in 20 healthy, physically active men and women with- and without autoregulation of applied BFR pressures using the Delfi Personalized Tourniquet device. Participants exercised with 60% of supine AOP in a 4 s per repetition (2 s concentric/2 s eccentric) cadence. Their results are in contrast to Jacobs et al. (Jacobs et al., 2023) as they found no differences in volume performed between BFR conditions nor in rate of perceived exertion and rate of perceived discomfort. However, they did note that autoregulation blunted the exercise-induced increases in central stiffness compared to both non-autoregulation [Mean difference, [MD] = 0.57 ± 1.12 m/s, 95% CI (0.05–1.09), *p* = 0.017, effect size [ES] = 0.51] and low-load exercise [MD = 0.63 ± 1.42 m/s, 95% CI (+0.04–1.3), *p* = 0.032, ES = 0.44], albeit with wide CIs. The non-autoregulated cuff condition also produced significantly greater increases in post-exercise muscle swelling and potentially muscle damage than the autoregulated condition as evidenced by greater muscle cross-sectional area (MD = 0.61 ± 1.03 cm^2^, 95% CI = 0.13–1.09, *p* < 0.01, ES = 0.59) and echo intensity [MD = 5.84 ± 8.89 au, 95% CI (1.67–9.99), *p* < 0.01, ES = 0.66]. While muscle damage was not directly sampled, prior research has indicated that post-exercise muscle swelling is a likely indicator of muscle damage, particularly if increased echo intensities along with larger muscle cross-sectional areas are observed (Damas et al., 2016). Of note, this study did not record any adverse events in either BFR condition throughout the study.

The divergent results on performance, rate of perceived exertion, rate of perceived pain and incidence of adverse events may be partially explained by differences in autoregulation capacity of the different BFR devices, participant characteristics, or type of exercise performed. Of potential relevancy is the practical observation that commercially available BFR devices vary in their capacity to provide quick adjustments to applied pressure during exercise, likely limiting conclusions about autoregulation to a particular device and not the feature itself. Future BFR research should specifically report the presence or absence of autoregulation given the preliminary body of research indicating it may impact BFR exercise responses.

### 2.3 Contour vs. straight cuff

**FIGURE 3:**
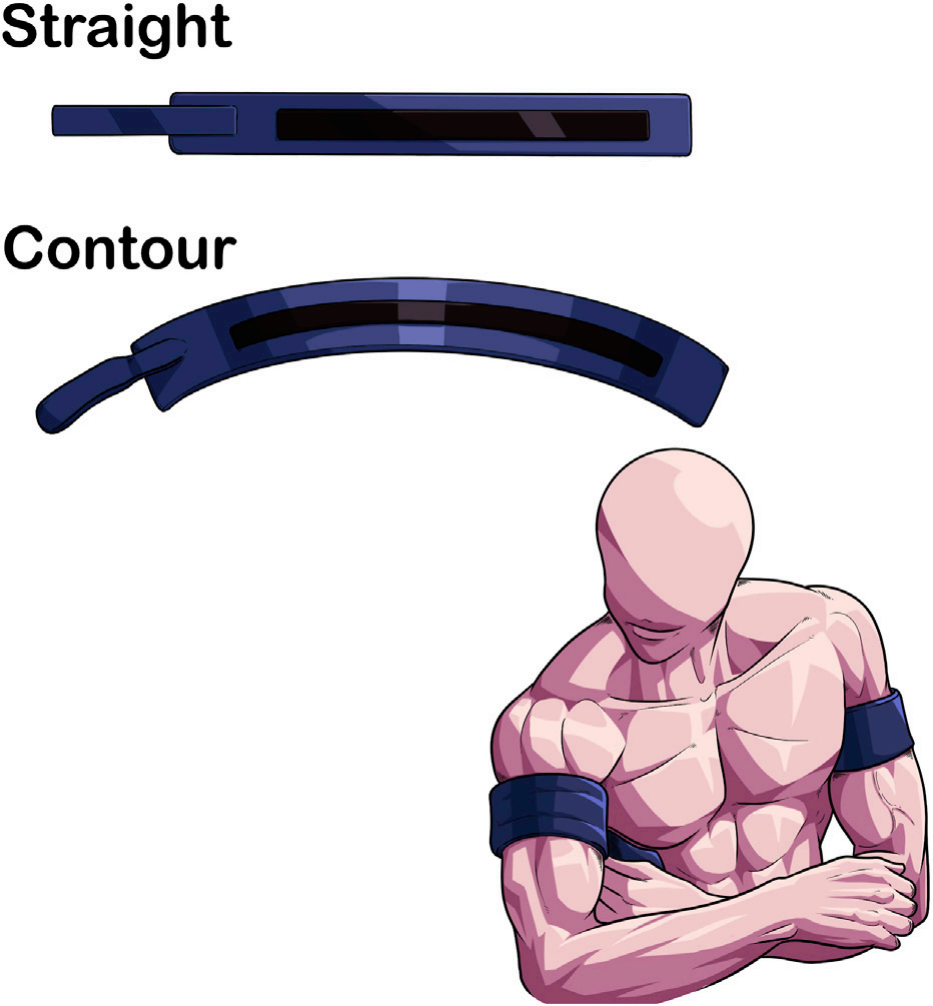
Differences in limb fit between contoured and straight BFR cuffs. contour cuffs provide a more secure fit due to the conical shape of the limb compared to a straight cuff. This may enhance the safety profile of BFR exercise.

Cuff shape has been shown to impact the amount of applied pressure needed to determine AOP (Figure 3) (Younger et al., 2004). Contour cuff shapes are longer at the top and shorter at the bottom, creating a tapered fit on the limb due to differences in diameter. Contoured cuffs also can be manufactured with variable contour shape, a design feature that allows for an even more secure fit to the limb as the device fastener apparatus can account for small differences in extremity size and shape (Tourniquet technology, 2017). Nonetheless, the difference in proximal to distal diameter of a contoured cuff reduces AOP slightly (∼−25.4 ± 16.1 mmHg measured with doppler ultrasound) compared to a straight cuff (e.g., cuff that is similar length on the top and the bottom) in the lower body (Pedowitz et al., 1993). Small differences were also noted in the upper body AOP (124.2 ± 10.5 mmHg vs. 128.5 ± 13.9 mmHg in contoured and straight cuffs, respectively), but are likely practically insignificant (Pedowitz et al., 1993).

Further, the occlusive stimulus may be different as straight cuffs are more likely to apply asymmetric pressures to the limb given the change in limb circumference proximally to distally in the extremities (Noordin et al., 2009). In populations where pressures during BFR exercise may want to be minimized to reduce the pressor response (Spranger et al., 2015), the use of a contoured cuff may be preferred to accommodate for the conical limb shape, particularly in the lower extremities. To date, no study has directly compared the acute and longitudinal responses to a BFR exercise regimen using cuffs of similar widths but varying in cuff shape, so this area of research is largely unknown.

### 2.4 Set pressure versus pressure applied to the limb

**FIGURE 4:**
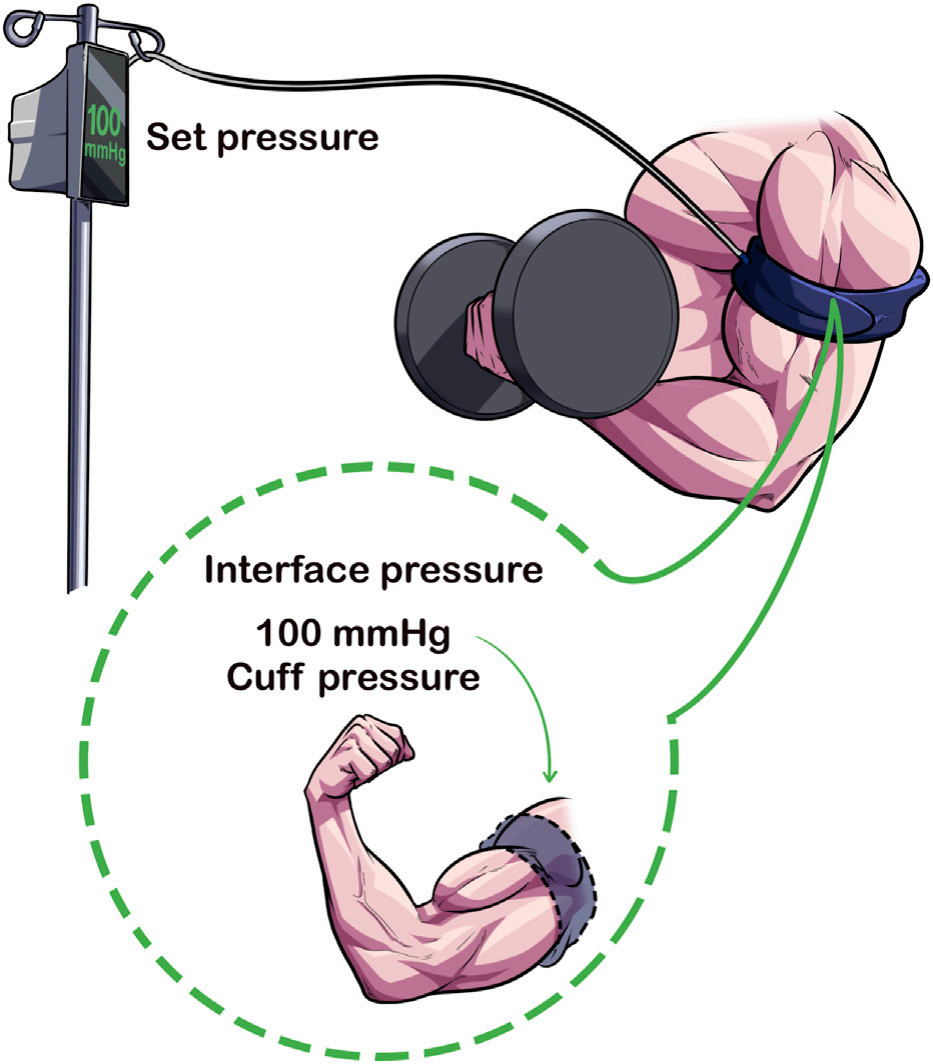
Set Pressure Versus Interface Pressure. The set pressure is the pressure that the pneumatic cuff is inflated to by the clinician/exerciser/researcher whereas the interface pressure is the amount of pressure applied to the limb from the cuff. Cuffs that can maintain a similar set and interface pressures may enhance acute safety of BFR exercise.

The pressure that is set for BFR (i.e., “the set pressure”) may not be the same pressure that is applied to the limb, known as the “interface pressure” (Figure 4) (Hughes et al., 2018). Hughes et al. (Hughes et al., 2018) showed that when the Delfi Personalized Tourniquet device (automatic autoregulated; cuff width = 11.5 cm; contoured cuff shape) was inflated to 40% and 80% AOP in a resting condition, the interface pressure was 8 ± 4 mmHg (95% CI: 16.84 to −0.17) and 9 ± 4 mmHg (95% CI: 16.80 to −0.32) lower than the set pressure (*p* < 0.05). When the manual cuff [Occlusion Cuff (The Occlusion Cuff LTD., Belfast, Ireland), cuff width = 8 cm; straight cuff shape] was inflated to similar relative pressures, the interface pressure was 20 ± 10 mmHg (95% CI: 39.16 to −1.40) and 37 ± 13 mmHg (95% CI: 62.12 to −11.88) lower than the set pressure (*p* < 0.05). Thus, despite personalizing the pressure to %AOP, the amount of applied pressure to the limb during resting conditions varied significantly between devices.

Preliminary results from Hughes et al. (Hughes et al., 2018) also indicated that cardiovascular and perceptual experiences were heightened in the manual cuff compared to the autoregulated cuff during exercise, with interface pressures greatly exceeding the clinical recommendation of ±15 mmHg applied pressure for safe tourniquet application (McEwen, 1981). Paradoxically compared to the lower interface pressures recorded during rest, the interface pressure compared to the set pressure during exercise was significantly elevated ranging between 37 ± 36 mmHg [95% CI: 33.79–108.01] in set 4 to 62 ± 35 mmHg (95% CI: 6.79–130.57) in set one; all *p* < 0.01]. In contrast, the Delfi Personalized Tourniquet device maintained the set and interface pressure during exercise and did not exceed ±15 mmHg in any of the four sets measured (*p* > 0.05). Elevations observed in the manual cuff over the Delfi Personalized Tourniquet device in rates of perceived exertion (e.g., 17 ± 2 vs. 15 ± 2 after set 4, 95% CI: 0.794–3.095, *p* < 0.01), rates of perceived pain (e.g., 8.3 ± 2.3 vs. 5.7 ± 2.0 after set 4, 95% CI: 1.359–3.808, *p* < 0.01) and mean arterial pressure 1-min post-exercise (11 ± 6 mmHg, 95% CI: 5.558–16.190, *p* < 0.01) may be at least partially explained by differences in the pressure applied to the underlying limb during exercise.

Despite setting AOP to a similar percentage based on the cuff (80% AOP), the comparison was not direct as cuff widths varied between devices, the Delfi Personalized Tourniquet device is autoregulated, and their cuff shapes varied. Insomuch as what’s currently known from the devices in the consumer market, the Delfi Personalized Tourniquet device has been shown to apply a pressure within measurement error and safe tourniquet use (±15 mmHg), ensuring a stimulus that is like the set pressure during exercise conditions. If possible, future studies should integrate measurements for determining interface pressures, particularly when novel devices are being investigated. Special attention should be paid to studies using lower (40%–50% AOP) pressures in their lower body interventions as this may impact the clinical relevance given lower pressures in this range have been shown to be ineffective at accelerating fatigue accumulation in BFR exercise (Cerqueira et al., 2021). If a cuff used in a lower pressure intervention was shown to be ineffective, researchers should determine if it was ineffective due to the parameters set (e.g., lower pressure) or inadequate cuff restrictive capabilities.

Lastly, in addition to cuff design features, interface pressure may be impacted by how snugly the cuff is applied, affecting pressure transmission to the limb by as much as 50% (Graham et al., 1993). It may be important for researchers to attempt to standardize a baseline level of tightness for everyone to reduce the impact of a too tightly or loosely fitting initial pressure. It also should be mentioned that cuff overlap impacts the applied pressure to the limb and has been recommended to be between 3–6 inches (Kumar et al., 2016). Values within this range likely apply a more uniform pressure to the underlying limb and may result in a more accurate interface pressure relative to the set pressure.

### 2.5 Presence/absence of an internal stiffener

A stiffener is a feature of a tourniquet that directs the pressure from the bladder onto the limb and helps maintain the cuff’s position when inflated to prevent slippage or skin pinching (McEwen et al., 2015; Tourniquet technology, 2017). The presence of an internal stiffener may impact the degree of AOP and/or the exerciser’s perceptual experiences during exercise as its presence increases the resistance to cuff deformation with muscular contraction (Tourniquet technology, 2017; McEwen et al., 2019). With respect to BFR exercise, no study has investigated the impact of an internal stiffener on cuffs with similar widths to determine its effect on acute- and longitudinal training outcomes. Future studies should determine its relevance with BFR exercise as more devices are being purchased and used in practice (Cuffe et al., 2022).

### 2.6 Bladder length—circumferential vs. partial circumferential

**FIGURE 5:**
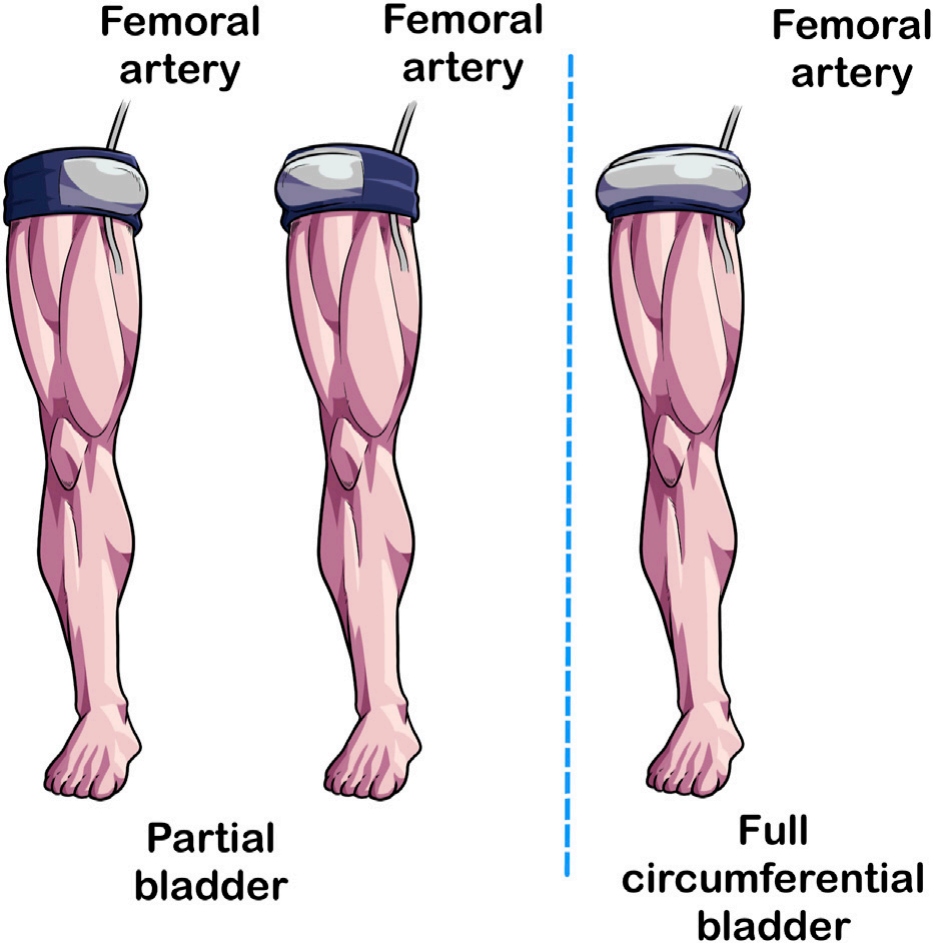
Partial circumferential versus circumferential bladder length. In traditional tourniquets, the bladder extends the length of the cuff (Right Image) whereas in some BFR cuffs, the bladder extends partially not covering the entirety of the length of the cuff (Left and Center Illustrations). Studies implementing BFR cuffs with partial circumference bladders should specify the position of the bladder because its placement may impact acute responses to BFR exercise.

The last cuff feature that can impact BFR exercise is the length of the bladder (Figure 5). In traditional tourniquets, the bladder circumferentially envelopes the limb (Kumar et al., 2016). In partial circumferential bladders, the bladder does not extend the length of the cuff, leaving areas without pneumatic pressure application that instead relies on compression from the sleeve of the device. As most, but not all [e.g., Airbands/SAGA Fitness Cuffs (VALD Health, VALD Pty Ltd., Newstead QLD, Australia)] BFR devices on the marketplace have circumferential bladders, little is known about the acute responses associated with differences in bladder length.

Currently, three studies exist utilizing partial circumferential bladders related to BFR (Spitz et al., 2020; Keller et al., 2023; Królikowska et al., 2023) but none of them have been used in the context of measuring acute- or longitudinal exercise responses. Two studies focused on methodological aspects of the partial bladder design (Spitz et al., 2020; Keller et al., 2023) and one investigated the impact of BFR on post-exercise joint position sense in recreational athletes (Królikowska et al., 2023). We want to briefly highlight the two studies (Spitz et al., 2020; Keller et al., 2023) investigating the methodological aspects associated with a partial bladder design and comment on their potential impact in practice and research design.

In a crossover within-subjects design (n = 32; 13 males), Spitz et al. (Spitz et al., 2020) showed that positioning the bladder on the outside of the thigh produced a greater AOP than when the bladder was positioned on the inside of the thigh (median difference of 13.56 mmHg, 95% CI: 7.29–19.84, Bayes factor [BF]_10_ = 437.52). In addition, agreement between bladder positions was worse with individuals with larger limb circumferences (r = 0.558, 95% CI: 0.24–0.74, BF_10_ = 42.863), highlighting the relative importance of standardizing the bladder position in research and practice with partial circumferential cuff designs. The difference in AOP between positions was attributed to the location of the femoral artery, the main conduit artery of the lower extremity. As the femoral artery is located anteromedially and not anterolaterally, the inside position required less pressure to occlude arterial inflow than when positioned anterolaterally. If bladder positioning impacts AOP in cuffs with partial circumferential bladder designs, this may have relevancy for clinical populations where limited applied pressure may enhance acute safety and/or longitudinal training responses. As Spitz et al. (Spitz et al., 2020) did not measure acute physiological and perceptual responses during exercise, it is unknown whether the positioning of the bladder and the magnitude of applied pressure has relevancy for BFR exercise.

Keller et al. (Keller et al., 2023) sought to validate the AOP algorithm used in a commercially available partial bladder BFR cuff system (Airbands) in the upper and lower extremities in 107 healthy males and females (n = 67 males). They compared the AOP given by the Airbands system with a gold standard doppler ultrasound assessment (using a circumferential bladder medical tourniquet [Tourniquet Touch TT20, VBM Medizintechnik GmbH, Sulz am Neckar, Germany]) in the seated position (both had 8 cm-widths). Of note, they did not specify where the bladder was positioned on the limb relative to the brachial and femoral artery with the Airbands cuff, only that it was standardized at identical positions during measurement for everyone. Their results indicated that the Airbands cuff provided considerable agreement with doppler ultrasound (125 ± 17 mmHg in the Airbands vs. 131 ± 14 mmHg in the doppler ultrasound assessment; mean difference = 7 ± 13 mmHg, 95% CI: 3–11) in the upper extremities. In the lower extremities, the Airbands cuff likely significantly underestimated AOP in 38 of 55 individuals (e.g., all had AOP of 270 mmHg) possibly due to limitations in the cuff compression technology that was unable to apply pressures greater than 270 mmHg. However, in a sub-group analysis of the 17 individuals with AOPs less than 270 mmHg, there was considerable agreement with doppler ultrasound (223 ± 14 mmHg for Airbands vs. 218 ± 23 mmHg for doppler ultrasound; mean difference = −5 mmHg, 95% CI: 17—8). Thus, it appears that in individuals whose limb circumferences are small, the Airbands cuff produces similar AOP values in the upper and lower extremities compared to a gold standard doppler ultrasound assessment using a circumferential bladder cuff. Limitations in the lower extremities on accurately predicting AOP may be of importance for future research in the lower body using the Airbands cuff as it will likely be unable to determine a personalized pressure for many individuals with larger thigh circumferences. Nonetheless, it should be acknowledged that this is not necessarily a safety issue (as the device likely cannot fully occlude the lower extremities in most individuals) but moreso a technological limitation given the reduced capability to standardize a restrictive stimulus.

Both studies provide preliminary insights into the ways in which a partial bladder system influences AOP that can guide future research. First, it appears that the bladder position matters with respect to the conduit artery, particularly in those with larger limb circumferences. Future studies using partial bladder cuff systems should specify where the bladder is relative to the conduit artery. Second, the commercially available Airbands BFR device is likely safe and valid to use for both the upper and lower extremities, but caution should be made with individuals that have larger thigh circumferences as AOP is likely under-estimated. Future studies using Airbands should monitor for lower extremity AOP values of 270 mmHg, as it indicates that the limb is likely too large to have AOP accurately determined. As such, this prevents a personalized pressure and will likely impact the magnitude of acute physiological and perceptual responses and potentially chronic training responses to BFR exercise. Last, no research exists investigating the responses of a partial bladder to exercise with BFR compared with a circumferential bladder inflated to the same relative pressure. As practitioners report using partial bladder systems in practice (Cuffe et al., 2022), understanding the impact of this cuff design on BFR exercise warrants future study.

## 3 Discussion

As discussed above, numerous cuff features may impact BFR exercise. While features like autoregulation appear to have some ability to modulate intra-exercise responses and potentially reduce adverse events, other features like bladder type (e.g., multi-chambered bladder systems) have the capacity to impact the ability to determine a personalized pressure. Importantly, while there are numerous ways to apply the BFR exercise stimulus (e.g., arbitrary pressures or %AOP), extrapolating acute responses using non-personalized pressure applications requires caution given the current body of evidence. Other cuff features such as bladder length (e.g., circumferential *versus* partial circumferential), presence/absence of an internal stiffener, and set/interface pressure are not widely studied and require further investigations into their potential relevancy in BFR given the existing body of research. Of note, no studies currently exist investigating the impact of an internal stiffener on determination of AOP or acute- or longitudinal responses to BFR exercise.

## 4 Conclusion

This manuscript attempted to contextualize the potential importance of infrequently reported BFR cuff features and hypothesize their potential impact on BFR training. As BFR continues to expand into practice, researchers should be aware of not only the importance of AOP assessment and its impact on BFR exercise responses, but of the ways that physiological responses may vary between cuffs despite standardization to %AOP. Cuffs that are unable to be standardized to a %AOP (e.g., multi-chambered bladder systems) may have clinical utility, but the current body of evidence on their efficacy is lacking and should be a focal area of future research—particularly if similar beneficial results are obtained with reductions in adverse events.

## Data Availability

The original contributions presented in the study are included in the article/Supplementary Material, further inquiries can be directed to the corresponding author/s.
